# Supplementary material for: Label-free concurrent 5-modal microscopy (Co5M) resolves unknown spatio-temporal processes in wound healing
Source: Commun Biol. 2021 Sep 6;4:1040. doi: 10.1038/s42003-021-02573-5 (PMC8421396; doi:10.1038/s42003-021-02573-5)
Supplement: Supplementary file 2 — Supplementary Information [file 42003_2021_2573_MOESM2_ESM.pdf]

Label-free concurrent 5-modal microscopy (Co5M) resolves unknown spatio-temporal processes in wound healing

SUPPLEMENTARY INFORMATION

Markus Seeger[1,2], Christoph Dehner[1,2], Dominik Jüstel[1,2], Vasilis Ntziachristos[1,2]

[1]Chair of Biological Imaging, Central Institute for Translational Cancer Research (TranslaTUM), School of Medicine, Technical University of Munich, Germany

[2]Institute of Biological and Medical Imaging, Helmholtz Zentrum München (GmbH), Neuherberg, Germany

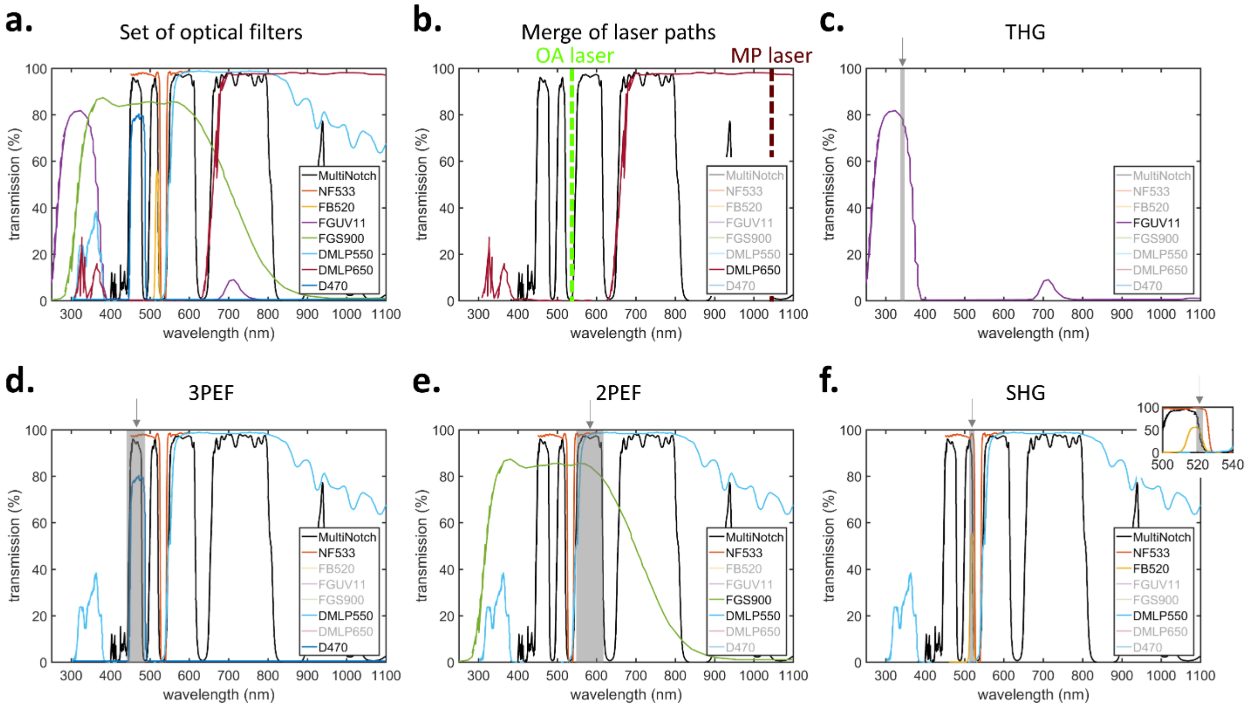

Supplementary Figure 1 ***Spectral merge of the multiphoton and optoacoustic excitation and detection. (a)*** *The combined set of optical filters installed for concurrent multimodal microscopy.* ***(b)*** *Spectral merge of the optoacoustic laser at 532 nm and the multiphoton laser at 1043 nm. Relevant optical filters for detecting* ***(c)*** *THG,* ***(d)*** *3PEF,* ***(e)*** *2PEF, and* ***(f)*** *SHG. Arrows indicate spectral position of the respective multiphoton signals; Transmission plots are extracted from the respective data sheets.*

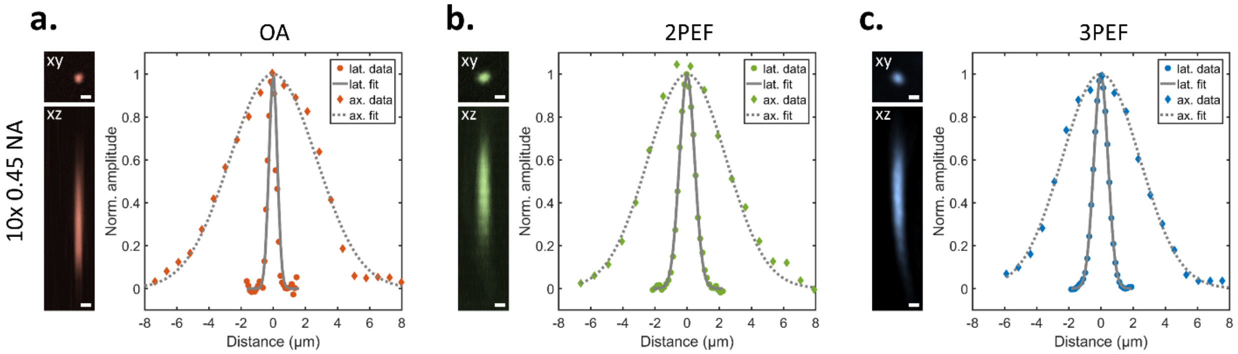

Supplementary Figure 2 ***Resolution determination of Co5M by imaging microspheres.*** *The achieved spatial resolution is characterized by imaging 200 nm black microspheres for OA and 100 nm fluorescent microspheres for 2PEF and 3PEF (2PEF and 3PEF is used to determine the resolution of the SHG and THG as other two- and three-photon modalities) with a 10x 0.45 NA objective.* ***(a)*** *xy- and xz-MAP images for OA using the 10x 0.45 NA objective; lateral (dots) and axial (diamonds) data profiles and lateral (line) and axial (dashes) Gaussian fits. Analogous depiction of* ***(b)*** *2PEF and* ***(c)*** *3PEF. Resolutions are determined to be 604.3 nm laterally and 6.324 µm axially for OA, 1104.0 nm laterally and 5.710 µm axially for 2PEF and SHG, and 1072.3 nm laterally and 5.667 µm axially for 3PEF and THG.*

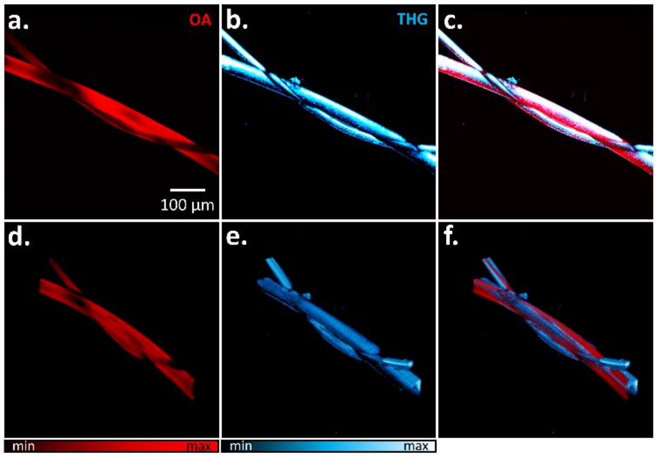

Supplementary Figure 3 ***Co-registration of the modalities using a suture phantom.*** *Co-alignment of the modalities using a suture phantom giving rise to OA and THG signals. 2D projections of the* ***(a)*** *OA and* ***(b)*** *THG measurement.* ***(c)*** *The overlay indicates negligible lateral offset among the modalities referenced to OA of 1.4 µm in +x and 1.2 µm in +y.* ***(d-e)*** *Analogous 3D projections of the signals.* ***(f)*** *3D overlay after correction of the chromatic aberration induced focal shift between the excitation wavelengths (-34.8 µm referenced to OA) and time-of-flight differences in OA signals originating from lateral offset positions.*

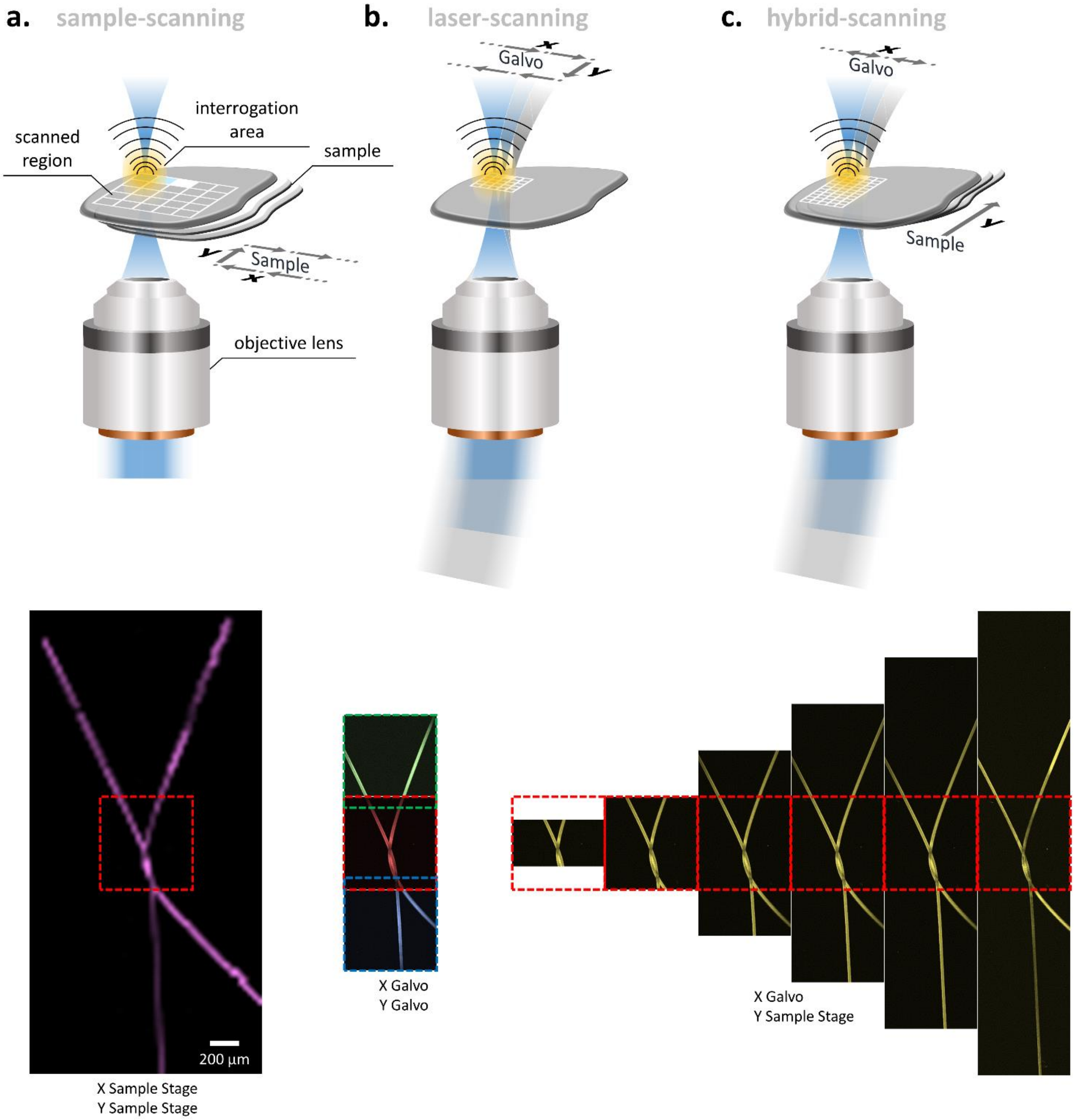

Supplementary Figure 4 ***Representative results of applying different Co5M scanning schemes by imaging black sutures with OA. (a)*** *Sample-scanning translates the sample in a stepwise manner through a stationary interrogation area and can image arbitrarily large FOVs for a coarse assessment of the specimens.* ***(b)*** *Laser-scanning raster-scans the optical focus across an area of max. ~630 µm edge length and is used for high-resolution high-speed imaging.* ***(c)*** *Hybrid-scanning continuously moves the sample and perpendicularly scans the optical focus bi-directionally along a line. Hybrid-scanning covers the specimen in stripes of width ~630 µm and arbitrary length and can be used for imaging large regions with high resolution. Arrows indicate movement direction of the respective parts. The central FOV of maximum size using laser-scanning is indicated as a red dashed box.*

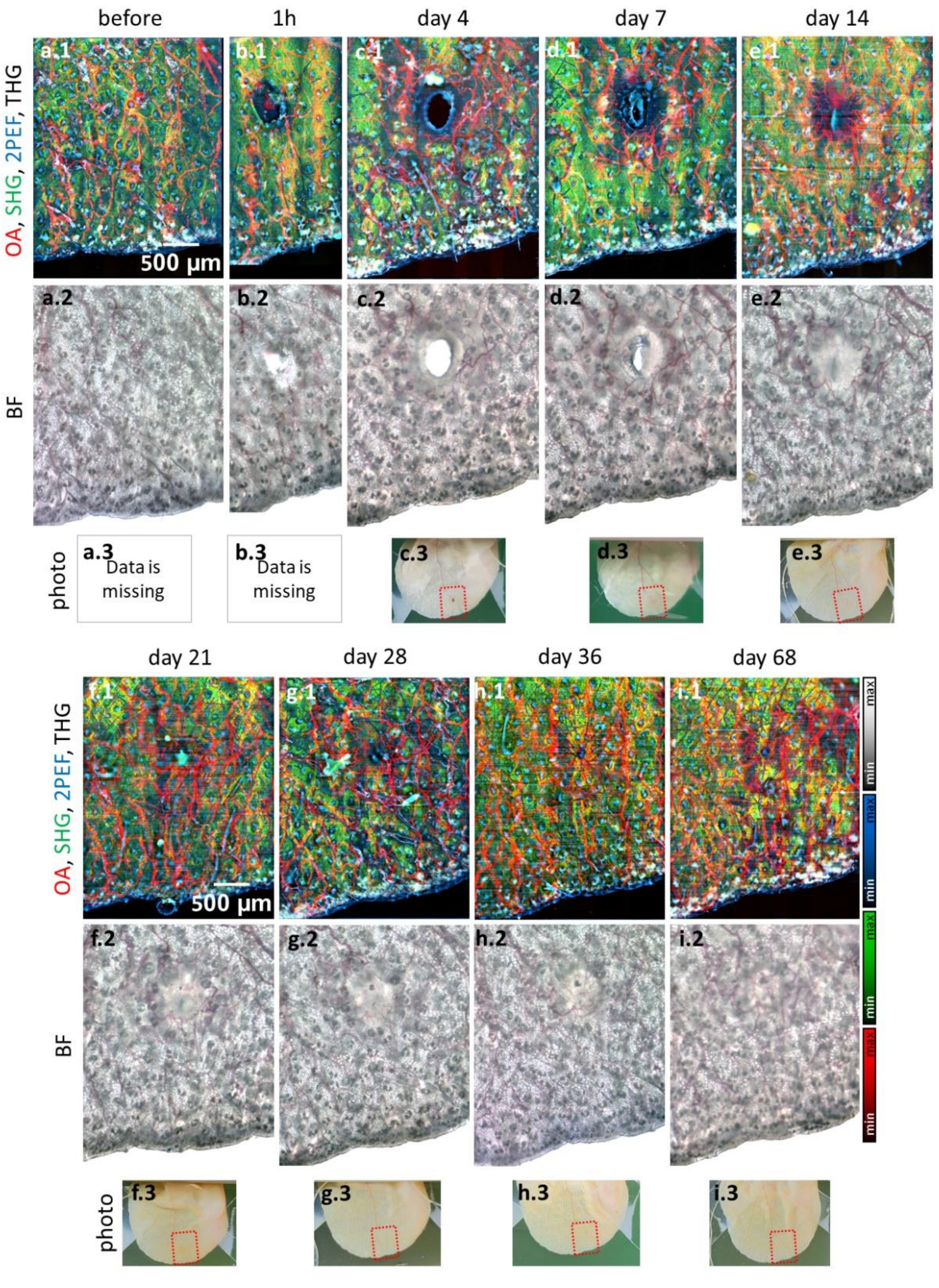

Supplementary Figure 5 ***Longitudinal imaging of the wound healing process in a mouse ear in vivo (mouse #2)*** *by label-free multimodal microscopy achieved using Co5M.* ***(a.1)*** *Multimodal image of the mouse ear before wound infliction and corresponding* ***(a.2)*** *brightfield microscopy image as well as* ***(a.3)*** *photograph of the ear. Analogous depiction of the ear at* ***(b.1-3)*** *1h,* ***(c.1-3)*** *4 days,* ***(d.1-3)*** *7 days,* ***(e.1-3)*** *14 days,* ***(f.1-3)*** *21 days,* ***(g.1-3)*** *28 days,* ***(h.1-3)*** *36 days, and* ***(i.1-3)*** *68 days after wound infliction.*

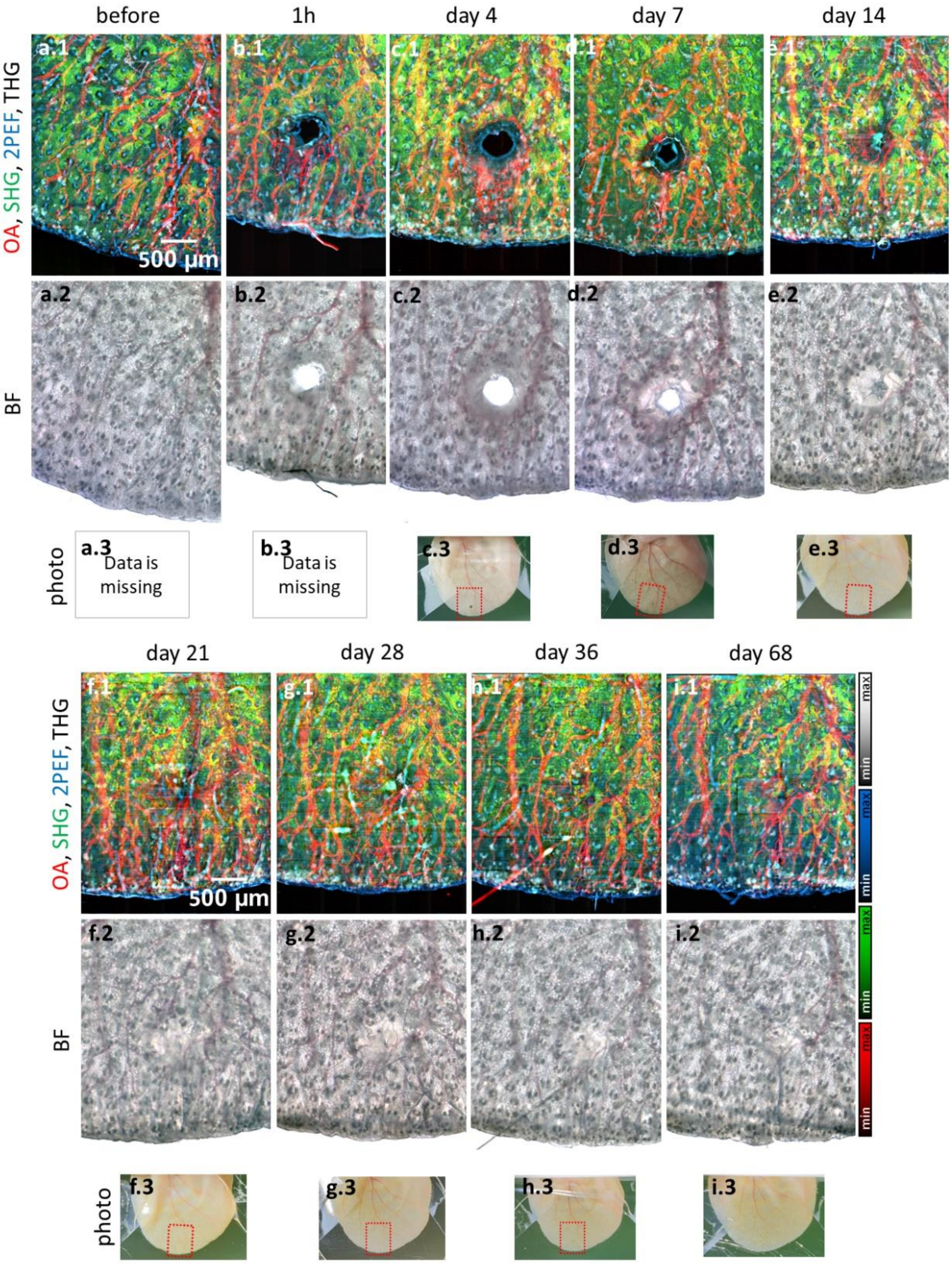

Supplementary Figure 6 ***Longitudinal imaging of the wound healing process in a mouse ear in vivo (mouse #3)*** *by label-free multimodal microscopy achieved using Co5M.* ***(a.1)*** *Multimodal image of the mouse ear before wound infliction and corresponding* ***(a.2)*** *brightfield microscopy image as well as* ***(a.3)*** *photograph of the ear. Analogous depiction of the ear at* ***(b.1-3)*** *1h,* ***(c.1-3)*** *4 days,* ***(d.1-3)*** *7 days,* ***(e.1-3)*** *14 days,* ***(f.1-3)*** *21 days,* ***(g.1-3)*** *28 days,* ***(h.1-3)*** *36 days, and* ***(i.1-3)*** *68 days after wound infliction.*

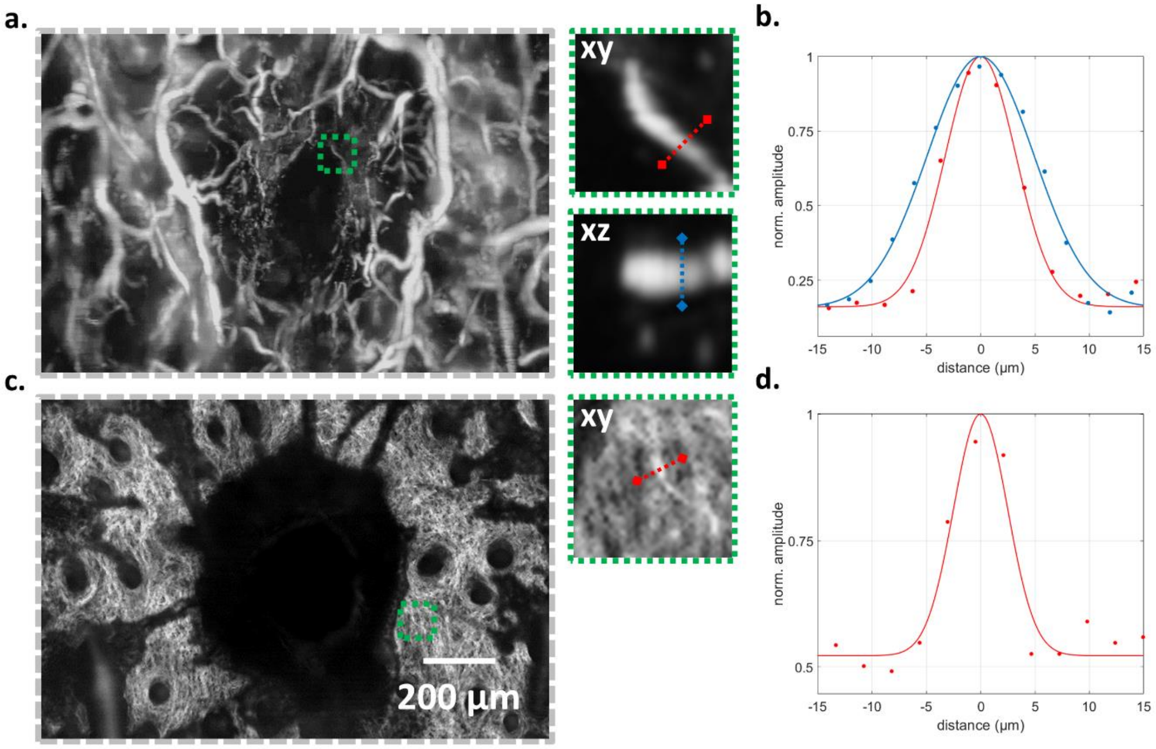

Supplementary Figure 7 ***Microstructures of tissue imaged with Co5M in vivo*** *using the OA (day 14) and SHG (day 4) images of mouse #1.* ***(a)*** *Zoom-in mouse ear vasculature imaged with OA and* ***(b)*** *Line profile of microvessel indicated with a red and blue line in (a) with a lateral diameter of 7.61 μm (Gaussian Fit, FWHM, R=0.9619) and an axial diameter of 11.57 μm (Gaussian Fit, FWHM, R=0.9834).* ***(c)*** *Zoom-in Mouse ear collagen network imaged with SHG and* ***(d)*** *Line profile of collagen fibril indicated with a red line in (c) with a diameter of 6.10 μm (Gaussian Fit, FWHM, R=0.9205).*

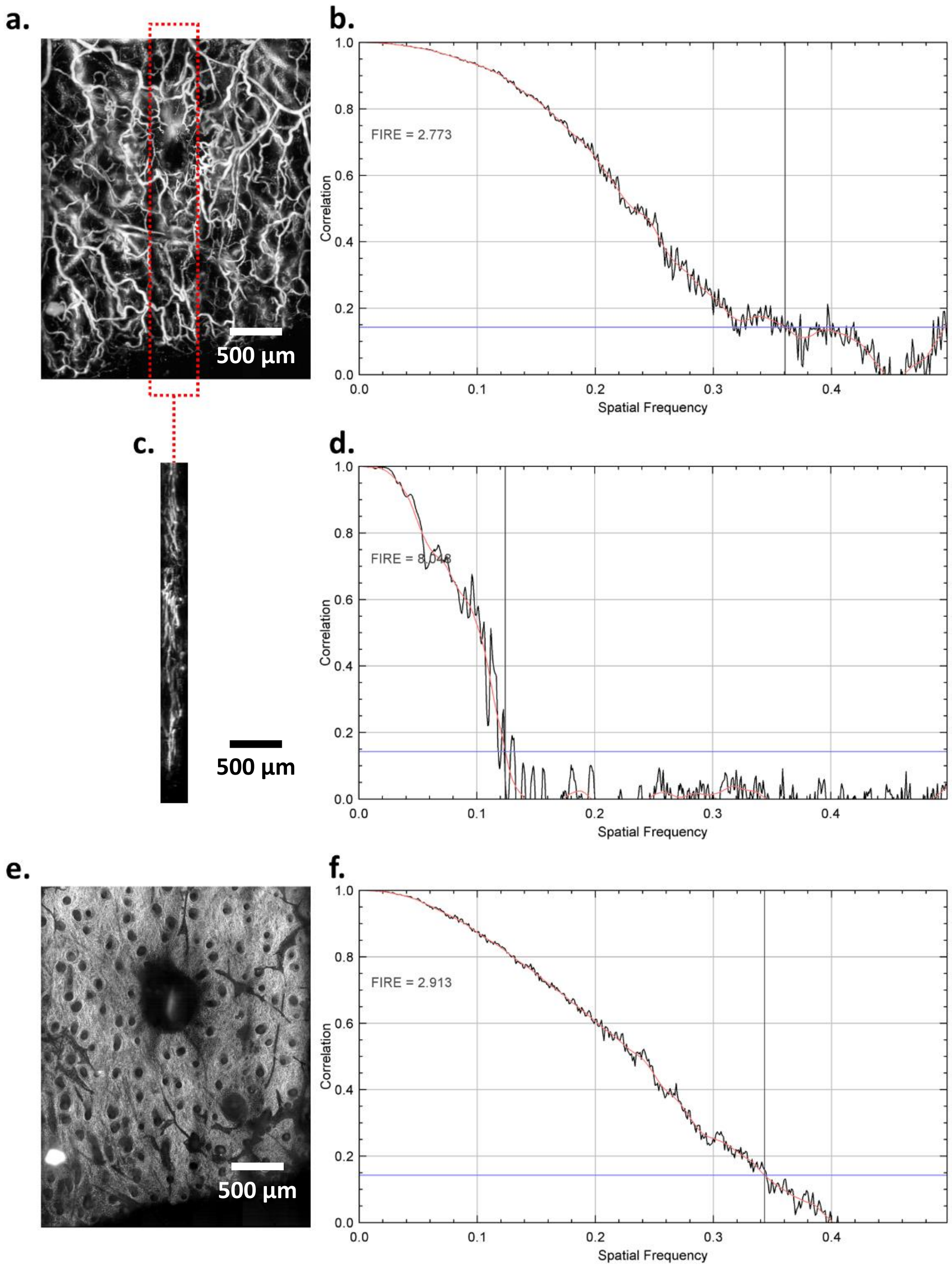

Supplementary Figure 8 ***Resolution determination of Co5M mouse tissue imaging in vivo*** *using the OA and SHG images of mouse #2 at day 14.* ***(a)*** *En-face OA image of microvasculature and corresponding* ***(b)*** *Fourier shell correlation with µm-scaled pixels reveal a Fourier Image Resolution (FIRE) of 2.773 µm.* ***(c)*** *Axial projection of central imaging FOV indicated with a red box in (a) and corresponding* ***(d)*** *Fourier shell correlation with µm-scaled pixels reveal a FIRE of 8.048 µm. (e) SHG image of extracellular collagen fibers and* ***(f)*** *Fourier shell correlation with µm-scaled pixels reveal a FIRE of 2.913 µm.*
