# Supplementary material for: Label-free concurrent 5-modal microscopy (Co5M) resolves unknown spatio-temporal processes in wound healing
Source: Commun Biol. 2021 Sep 6;4:1040. doi: 10.1038/s42003-021-02573-5 (PMC8421396; doi:10.1038/s42003-021-02573-5)
Supplement: Supplementary file 3 — Description of Additional Supplementary Files [file 42003_2021_2573_MOESM3_ESM.pdf]

Description of Additional Supplementary Files

File name: Supplementary Data 1.

Description: Source data for the charts shown in Fig. 4a-c and h-o capturing Co5M's spatio-temporal assessments of the wound healing process.
